# The Novel Functions of High-Molecular-Mass Complexes Containing Insulin Receptor Substrates in Mediation and Modulation of Insulin-Like Activities: Emerging Concept of Diverse Functions by IRS-Associated Proteins

**DOI:** 10.3389/fendo.2015.00073

**Published:** 2015-05-26

**Authors:** Fumihiko Hakuno, Toshiaki Fukushima, Yosuke Yoneyama, Hiroyasu Kamei, Atsufumi Ozoe, Hidehito Yoshihara, Daisuke Yamanaka, Takashi Shibano, Meri Sone-Yonezawa, Bu-Chin Yu, Kazuhiro Chida, Shin-Ichiro Takahashi

**Affiliations:** ^1^Department of Animal Sciences, Graduate School of Agriculture and Life Sciences, The University of Tokyo, Tokyo, Japan; ^2^Department of Applied Biological Chemistry, Graduate School of Agriculture and Life Sciences, The University of Tokyo, Tokyo, Japan; ^3^Laboratory of Biomedical Chemistry, Basic Life Sciences, Institute of Biomedical and Health Sciences, Hiroshima University, Hiroshima, Japan; ^4^Department of Biological Sciences, Faculty of Bioscience and Biotechnology, Tokyo Institute of Technology, Kanagawa, Japan; ^5^Laboratory of Protein Metabolism, Tokyo Metropolitan Institute of Medical Science, Tokyo, Japan

**Keywords:** insulin-like growth factor, insulin, insulin receptor substrate, IRS-associated protein, IRSome, cancer

## Abstract

Insulin-like peptides, such as insulin-like growth factors (IGFs) and insulin, induce a variety of bioactivities, such as growth, differentiation, survival, increased anabolism, and decreased catabolism in many cell types and *in vivo*. In general, IGFs or insulin bind to IGF-I receptor (IGF-IR) or insulin receptor (IR), activating the receptor tyrosine kinase. Insulin receptor substrates (IRSs) are known to be major substrates of receptor kinases, mediating IGF/insulin signals to direct bioactivities. Recently, we discovered that IRSs form high-molecular-mass complexes (referred to here as IRSomes) even without IGF/insulin stimulation. These complexes contain proteins (referred to here as IRSAPs; IRS-associated proteins), which modulate tyrosine phosphorylation of IRSs by receptor kinases, control IRS stability, and determine intracellular localization of IRSs. In addition, in these complexes, we found not only proteins that are involved in RNA metabolism but also RNAs themselves. Thus, IRSAPs possibly contribute to modulation of IGF/insulin bioactivities. Since it is established that disorder of modulation of insulin-like activities causes various age-related diseases including cancer, we could propose that the IRSome is an important target for treatment of these diseases.

## Introduction

### Bioactivities of insulin-like peptides

Insulin-like peptides, such as insulin-like growth factors (IGF-I and IGF-II) and insulin, are a family of peptide hormones that are highly homologous in structure and function. Both IGFs and insulin exert a variety of bioactivities that mainly increase anabolism and decrease catabolism.

Insulin-like growth factor-I is produced in a wide range of tissues including liver, a major IGF-producing tissue. Throughout life, IGF-I levels are positively regulated by growth hormone and insulin and negatively controlled by glucocorticoid and malnutrition (1). Meanwhile, IGF-II exhibits its highest levels in the fetal stage declining after birth. Both IGFs are constitutively secreted and thus do not show apparent diurnal variation. IGFs exert biological effects on most cell types [except for liver and fat tissues, in which IGF-I receptor (IGF-IR) declines after birth]. Therefore, IGFs can act by autocrine and paracrine mechanisms as well as by endocrine mechanisms. Many studies have analyzed bioactivities of IGFs using a variety of cell types including primary cultured cells and established cell lines, and have revealed that IGFs exert relatively chronic effects including induction of cell proliferation, differentiation, survival and migration, and maintenance of cell functions (2). Together with other reports (3), IGFs are believed to be indispensable for growth and development.

One of the characteristics of insulin is that this hormone plays a major role in metabolic actions (4–6). In response to elevated blood glucose levels, insulin is transiently secreted from pancreatic β cells, and it promotes glucose uptake and glycogen synthesis and inhibits glycogenolysis and gluconeogenesis in the target tissues such as muscle, adipose, and liver. In addition to these actions involved in glucose homeostasis, insulin also promotes uptake of amino acids, protein synthesis, and lipid metabolism.

These insulin-like activities are well regulated by a variety of hormones, growth factors, and cytokines under various physiological conditions (7–10). The fine regulation makes normal follicle development, implantation, development, growth, maturation, metabolic control, and aging possible (3, 11). Whenever dysregulation of insulin-like activities is induced, growth disorders and many types of age-related diseases including diabetes, cancer, neurodegenerative disease, arteriosclerosis, and osteoporosis develop (12–14). Especially, in the past several decades, evidence has accumulated that deregulation of insulin-like activities is involved in diseases including cancer. Using many types of cancer cells and genetically engineered mice, it has been demonstrated that IGFs and insulin promote cancer cell proliferation, motility, invasion, and survival, and augmentation of these activities is proposed as a mechanism for cancer development, growth, and metastasis (15–17).

### Signal transduction of insulin-like peptides

Insulin-like peptides transduce signals by binding to IGF-IR and insulin receptors (IR) on the plasma membrane of target cells. IGF-IR and IR are similar to each other and belong to the receptor tyrosine kinase family. These receptors form disulfide-linked hetero-tetramers composed of two α-subunits and two β-subunits, which possess extracellular domains interacting with hormones and intracellular domains with tyrosine kinase activity. IGFs bind to IGF-IR with high affinity, but to IR with low affinity. In the same manner, insulin binds to IR with high affinity, whereas to IGF-IR with low affinity. Moreover, IGF-IR forms hybrid receptors with IR in cells that equally express both IGF-IR and IR. These hybrid receptors are shown to possess high affinity for IGF-I (18). IR exists as two isoforms, IR-A and IR-B, which result from alternative splicing of IR pre-mRNA. IR-B mRNA includes exon 11, whereas IR-A mRNA lacks exon 11 that encodes the 12 amino-acid region of the C-terminus of the α-subunit. IR-B binds to insulin, whereas IR-A binds to not only insulin but also IGF-II. Because IR-A binds to IGF-II and is highly expressed in several cancer cells and tissues, it is likely to be involved in oncogenic transformation (19).

**Figure 1:**
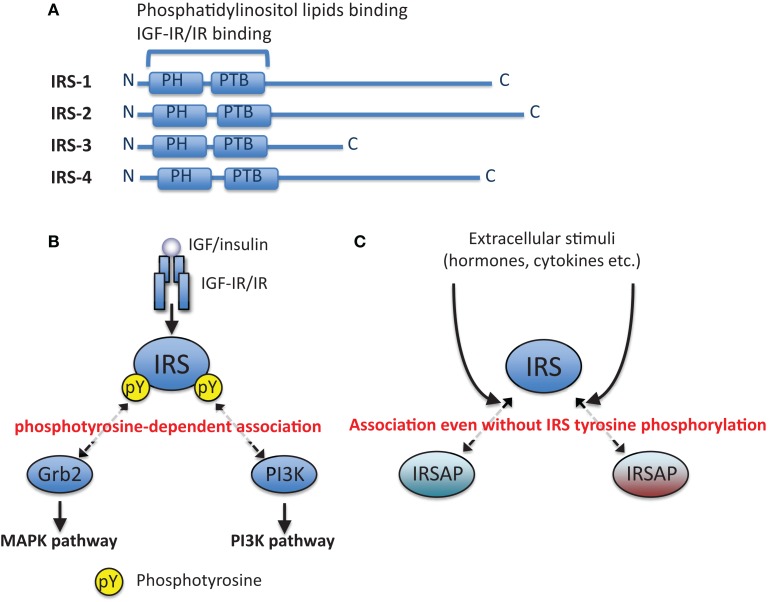
**Phosphotyrosine-dependent and -independent association of IRS with other proteins**. **(A)** Schematic illustration of IRS-1, IRS-2, IRS-3, and IRS-4. **(B)** Canonical IGF/insulin signaling pathways. IRSs are tyrosine-phosphorylated by IGF-IR/IR tyrosine kinase, and then they are associated with downstream signaling molecules in a phosphotyrosine (pY)-dependent manner. **(C)** IRSs are associated with other proteins (IRSAPs) even without their tyrosine phosphorylation, and this interaction can be changed by extracellular stimuli in addition to IGF/insulin stimulation.

Binding of ligands to their receptors (IGF-IR and IR) on the plasma membrane activates their receptor tyrosine kinase activities. The activated receptors tyrosine-phosphorylate themselves (auto-phosphorylation) and their specific substrates, some of which are insulin receptor substrate (IRS) proteins (Figure 1A). Tyrosine-phosphorylated IRSs are recognized by Src-homology (SH) 2-containing proteins (Figure 1B), one of which is phosphoinositide 3-kinase (PI3K). Phosphorylation of phosphoinositide in membranes by PI3K results in activation of serine/threonine kinase Akt. The activated Akt phosphorylates many substrates to promote various bioactivities. The other signaling molecule recognizing tyrosine-phosphorylated IRSs is Grb2. Grb2 is reported to form a complex with SOS to activate small GTPase Ras. The activated Ras induces the MAPK/ERK pathway leading to mitogenic activities (20). IRSs are associated with other proteins even without their tyrosine phosphorylation (Figure 1C), as described in detail below.

### Insulin-like peptides and cancer

**Figure 2:**
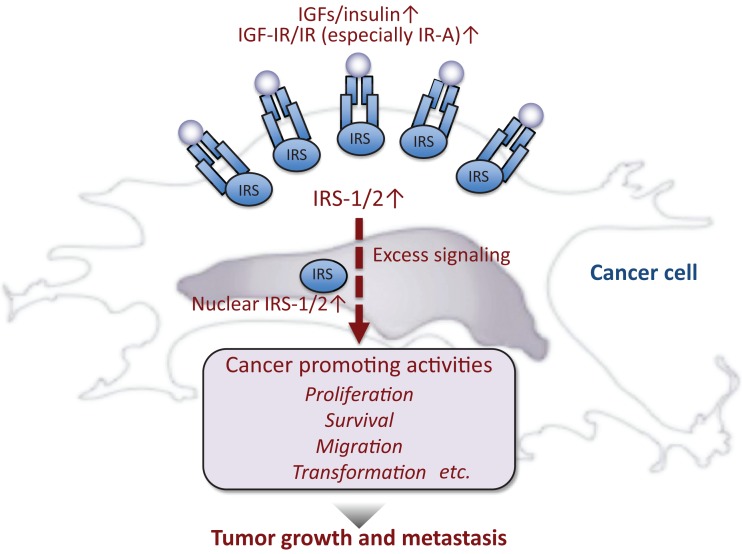
**Insulin-like activities and cancer**. In general, cancer cells produce or are exposed to high levels of insulin-like peptides and/or IGF/insulin signals are enhanced in these cells, resulting in maintenance of cancer phenotypes.

Accumulated evidence suggests that excess IGF/insulin activity contributes to cancer development (Figure 2). IGFs are often produced in cancer cells and/or the surrounding stromal cells. IGF-IR (21, 22) and IR (especially IR-A) (22–25) are also overexpressed in many cancer cells. In cell cultures, IGF/insulin promotes proliferation, survival, and migration of various cancer cells. IGF is also known to mediate the transforming action of many oncogenes (26). Recently, IGF/insulin is proposed to play roles in the maintenance of cancer cell stemness (27) as well as cancer metabolism (28).

Genetic and pharmacological manipulation clearly shows important roles of IGF/insulin and their receptors not only in cultured cancer cells but also in mouse tumor models. For example, injection of neutralizing antibody against IGF-I and IGF-II into mice suppressed the development of bone tumors (29, 30) and liver metastasis of colorectal cancers (31). Liver-specific IGF-I knockout mice showed delayed-onset of chemically and genetically induced mammary tumors (32), and neutralizing antibody against IGF-IR and IGF-IR kinase inhibitor showed anti-tumor effects in mouse xenograft models (33–35). These studies demonstrate roles of IGFs and IGF-IR in tumorigenesis and following studies show roles of insulin and IR. IR knockdown inhibited breast cancer cell proliferation, angiogenesis, and metastasis in nude mice (36), and pancreatic β cell-specific IR knockout impaired tumorigenesis in genetic mouse models with pancreatic neuroendocrine tumor (37). Moreover, combined action of IGF-IR/IR was also investigated. Pharmacological inhibition of both IGF-IR/IR kinases inhibited the growth of tumors transplanted from constitutively active IGF-IR-transgenic mice (38). The dual inhibitor also reversed tumor-promoting effects of type-2 diabetes in breast cancer mouse models (39). An antibody with neutralizing activity against both IGF-IR and IGF-I/insulin-hybrid receptors showed more potent anti-tumor effects than antibodies targeting only the IGF-IR or hybrid receptors in xenograft models (40). The combination of IR gene deficiency and IGF-IR-neutralizing antibody treatment suppresses tumor growth in a transgenic mouse model of pancreatic neuroendocrine carcinogenesis. However, the IGF-IR antibody does not significantly impair tumor growth in the model with the intact IR gene (37).

In humans, population-based studies show that high circulating IGF-I levels are associated with the risk of cancer development (41), and hyperinsulinemia is related to poor cancer prognosis (42, 43). Recently, a potential clinical benefit of IGF-IR-neutralizing antibodies was reported, but it was limited to a small subset of cancers [Ewing’s sarcoma (44), non-small cell lung carcinoma (45), and some other chemotherapy-resistant solid tumors (46)]. The failure of IGF-IR-targeted therapy in many other studies points out the putative importance of combinatory target therapy toward IGF-IR and IR (47).

## Insulin Receptor Substrates

### Characteristics and physiological significance of IRSs

The genes encoding IRS proteins are evolutionarily conserved through the animal kingdom, from worm to human, and a single or multiple IRS paralogous genes are found within a species. In vertebrates, three to four types of genes encoding structurally related IRS proteins (IRS-1, -2, -3, and -4) have been found in many species (48, 49). From previous reports and current genome database analyses, the human genome harbors IRS-1, -2, and -4 genes, but it apparently lost the IRS-3 gene during evolution though the mouse genome contains all four IRS genes. It is noteworthy that the most analyzable vertebrate genomes from fish to primates contain both IRS-1 and -2 genes (http://www.ensembl.org/index.html), implying roles for these genes in the regulation of fundamental physiological events conserved throughout chordate evolution. In invertebrate model species, the *chico* in fly and the *ist-1* in worm are known as functional IRS orthologous genes (50, 51).

Common structural features of IRS proteins are the pleckstrin homology (PH) and phosphotyrosine binding (PTB) domains in the amino-terminal region (Figure 1A). Since IRSs lack any enzymatic activities in their primary sequences, they are regarded as adaptor or docking proteins that mediate signaling. As we explained above, the IRSs interact with tyrosine-phosphorylated IGF-IR/IR through the PTB domains, and the tyrosine residues in IRSs are subsequently phosphorylated via the receptor tyrosine kinases. IRS-2, which has a unique structural feature, also interacts with the tyrosine kinase domain of IRs through its specific region called as KRLB region (52, 53). The tyrosine-phosphorylated IRS recruits major IGF/insulin signaling molecules to the membrane through its binding to membrane with its PH domain, which leads to the activation of PI3K–Akt and/or Ras–MAPK signaling cascades. Consequently, IRS serves a major branching node of IGF/insulin signaling (Figure 1B).

For IGF/insulin-dependent PI3K–Akt activation, several specific tyrosine residues in the binding motif of the SH2 domains of class I PI3K p85 regulatory subunit (YxxM; where X is any amino acid) are typically important. The phosphotyrosine residues at 608, 628, 658, and 939 in IRS-1 (54–56) and at 649, 671, 734, and 814 in IRS-2 were demonstrated to be involved in PI3K p85 binding (57). The tyrosine 891 in IRS-1 is known to be the Grb2-binding site phosphorylated by IGF/insulin-stimuli, which is involved in Ras–MAPK cascade activation (58). In addition, the serine/threonine phosphorylation and the post-translational modifications of lysine residues in IRS also play major roles in fine tuning the IGF/insulin signaling. S6K phosphorylates serine/threonine residues of IRS, which in turn leads to the degradation of IRS protein and attenuation of IGF/insulin signaling (20, 59). It has been reported that S6K directly phosphorylates IRS-1 on multiple serine residues including IRS-1 serine 307 to inhibit insulin signaling, and these serine phosphorylations were increased in adipose tissues in obese mice (60). By contrast, a recent study shows that IRS-1 serine 307 phosphorylation promotes insulin sensitivity (61). The serine/threonine phosphorylation of IRSs could be complex but an important clue for deciphering the pathology of IGF/insulin signaling. The ubiquitination on lysine residues in IRS causes degradation of the IRSs (59, 62, 63). Also, the IRS-2 acetylation on its lysine residue decreases IGF-induced MAPK signaling in neuron and protects neurons from oxidative damage induced cell death (64).

The physiological significance of IRS proteins has been studied by using gene knockout mice (65–80). The study using the *Irs-1* gene null mice or using the cells from the knockout animal have established the notion that *Irs-1* plays crucial roles in growth and development while *Irs-2* is indispensable for maintaining systemic glucose metabolism rather than mediating signals for body growth (65, 75). The physiological roles of IRSs are not limited to glucose metabolism and growth (50, 69, 72, 73, 78, 81–87). IRS-1 maintains vascular health, and IRS-1 and IRS-2 governed bone turnover and adipocyte differentiation (72, 78). The massive increase in hepatic IRS-2 under protein malnutrition condition suggests that IRS-2 coordinates liver function with changing amino-acid nutrition *in vivo* (83). It is also noteworthy that loss of the *Irs* gene in both rodent and fly models resulted in extended lifespan (50, 85–87). Intriguingly, it has been recently reported that IRS-1 interacts with RNA molecules (88, 89). These facts underscore the multifunctional characteristics of IRS proteins in various pathophysiological contexts.

### IRSs and cancer

IRSs contribute to in cancer development (Figure 2). High expression levels of IRS-1/2 are reported in various types of cancer cells (90). In addition, intense tyrosine phosphorylation of IRS-1 is found in a variety of solid tumors (91). Exceptionally, downregulation of IRS-1 is found in advanced breast cancer (92) and non-small cell lung cancer (93). From the clinical point of view, it should be noted that IRSs are often increased by the treatment of cancer cells with anti-cancer drugs targeting signaling pathways downstream of IRSs (94, 95). The possible underlying mechanism is that the drug-induced inhibition of the downstream signaling suppresses the negative feedback regulation that reduces IRS levels, thereby increasing IRS levels (96). This phenomenon may decrease the anti-cancer activity of the drugs. Interestingly, treatment of mice with novel compounds that promote IRS degradation significantly inhibited the growth of melanoma, ovarian, and prostate cancers (95).

Many studies have shown roles for IRS-1/2 in cancer development. In various cancer cells, overexpression/knockdown experiments indicate that IRS-1/2 promotes cell proliferation, survival, migration, and/or transformation (90, 94, 97–103). Transgenic mice with tissue-specific IRS-1/2 overexpression showed increased tumorigenesis and metastasis (104, 105). Knockout mice studies also reveal that IRS-1 deficiency decreases incidence and growth of several tumors (106, 107). IRS-2 deficiency suppresses metastasis (108, 109). Taken together, many studies indicate that IRS-1 and IRS-2 play redundant roles in cancer development, but some reports clearly show their distinct roles: IRS-1 tends to promote cancer proliferation whereas IRS-2 preferentially promotes cell migration (104). Moreover, in some cases, IRS-1 unexpectedly functions as a negative factor in cancer development (110–113). Roles of IRS-1/2 in cancer development may be dependent on tissues, oncogenic stimulations, and/or environmental factors.

Mechanistically, it is widely accepted that IRSs can regulate proliferation, survival, and migration of cancer cells through the activation of the downstream PI3K pathway and MAPK pathway. One of the still unsolved issues is how IRS-1 and IRS-2 play distinct roles in some models. Another issue is the molecular mechanism underlying IRS-promoted cell transformation. In addition, recent studies highlight nuclear IRSs observed in cancer, and their interaction with several nuclear proteins (114). In order to reveal mechanisms of IRS-driven cancer development, it is becoming more important to study proteins that interact with IRSs in isoform-specific and context-dependent manners.

## High-Molecular-Mass Complexes Containing IRSs

As described above, tyrosine phosphorylation of IRSs are important for transmission of IGF/insulin signals (Figure 1B). However, recently, we have reported that IRSs interact with various proteins even without IRS tyrosine phosphorylation (Figure 1C). Such IRS-associated proteins were referred to here as IRSAPs and their function of IRSAPs were reviewed below.

### Roles of proteins co-immunoprecipitated with IRSs in modulation of tyrosine phosphorylation-dependent signals of insulin-like peptides

We had previously reported that pretreatment of FRTL-5 thyrocytes with thyrotropin (TSH) or other cAMP-generating agents potentiated IGF-dependent tyrosine phosphorylation of IRS-2, leading to enhancement of cell proliferation in response to IGF-I (115). To investigate the mechanisms in the potentiation of tyrosine phosphorylation of IRS-2, we performed *in vitro* tyrosine phosphorylation assays. IRS-2 was prepared by immunoprecipitation using anti-IRS-2 antibody from cells treated with or without dibutyryl cAMP, and subjected to *in vitro* tyrosine phosphorylation assay using IGF-IR as the tyrosine kinase. Tyrosine phosphorylation by the IGF-IR of IRS-2 prepared from FRTL-5 cells treated with dibutyryl cAMP was higher than IRS-2 prepared from control cells (116), suggesting that long-term cAMP stimulus increased the availability of IRS-2 as a substrate to be phosphorylated more intensely by IGF-IR kinase. In addition, when the immunoprecipitated IRS-2 was washed with a high-salt buffer solution to dissociate protein components prior to performing the phosphorylation reaction, the cAMP-induced potentiation of tyrosine phosphorylation of IRS-2 was abolished (116). These results indicated that protein components co-immunoprecipitated with IRS-2 are necessary for the potentiation of tyrosine phosphorylation of IRS-2 by IGF-IR kinase in FRTL-5 cells. We also observed that the IRS-1 immunoprecipitated from tumor necrosis factor (TNF)-α-treated cells was little phosphorylated by IR tyrosine kinase *in vitro* and protein components co-immunoprecipitated with IRS-1 are necessary for TNF-α-induced decreases in the availability of IRS-1 to receptor tyrosine kinase (116). Based on these results, we concluded that proteins associated with IRSs play important roles in the modulation of IRS tyrosine phosphorylation by IGF-IR or IR.

### Forming high-molecular-mass complex containing IRSs

We then analyzed the molecular mass of complexes containing IRSs in several types of cells without IGF-I/insulin stimulation. Blue native-polyacrylamide gel electrophoresis (BN-PAGE) in which protein complexes are separated under native conditions was used, followed by immunoblotting with anti-IRS-1 antibody. In 3T3-L1 adipocytes, IRS-1 was detected at approximately 400–1,300 kDa (mainly 400 kDa) (116). In MCF-7 breast cancer cells, IRS-1 was detected at 400 and 1,300 kDa (88). We also analyzed the molecular mass of complexes containing IRS-2. In FRTL-5 thyroid cells, IRS-2 was detected at 400 kDa, and in MCF-7 cells IRS-2 was detected at 500, 800, and 1,300 kDa (88, 116). Given that IRS-1 and IRS-2 are usually detected at 150–180 kDa in SDS-PAGE, these results suggest that IRSs form high-molecular-mass complexes even without IGF/insulin stimulation. The molecular mass of the complexes differs with cell types and IRS isoforms.

Interestingly, the molecular mass changes not only with IGF-I/insulin stimulation but also with stimulation by other cytokines/hormones that modulate IGF-I/insulin signaling (Figure 1C). For example, stimulation of FRTL-5 cells with TSH or other cAMP-generating agents potentiates IGF-I-dependent IRS-2 tyrosine phosphorylation and cell proliferation (115), and cAMP stimulation increases the molecular mass of complexes containing IRS-2 from 400 kDa up to 800 kDa (116). It is possible that some molecules that modulate IRS-mediated signaling associate/dissociate with the complexes in response to stimulation by various cytokines/hormones, as described below. These complexes were referred to here as IRSome.

### Functions of proteins in the IRSome

**Figure 3:**
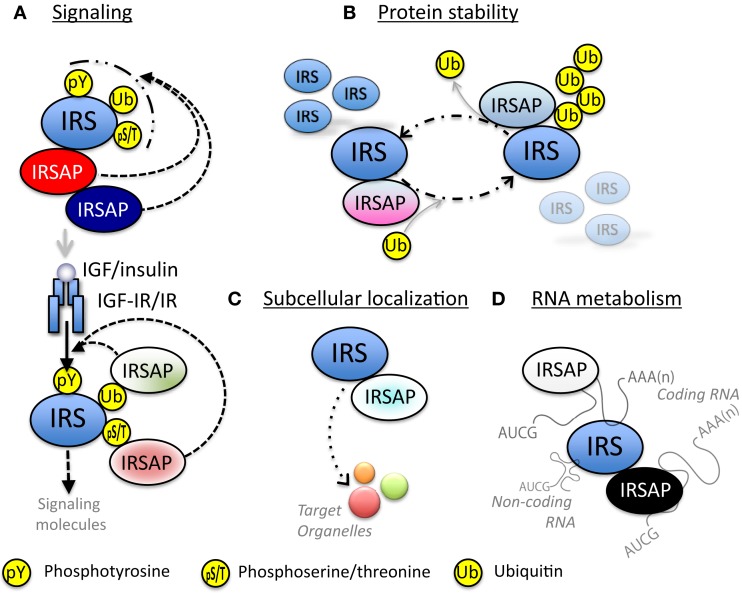
**Possible functions of IRS-associated proteins (IRSAPs)**. **(A)** IRS-associated proteins modulate IGF/insulin signal intensity through various mechanisms. Some IRSAPs regulate post-translational modification of IRSs. Other IRSAPs interact with IRSs through recognition of post-translationally modified IRSs. These modification and/or protein-interaction regulate the availability to IGF-IR or IR. **(B)** IRS-associated proteins control stability of IRSs proteins. **(C)** IRS-associated proteins transport IRSs to specific organelles. **(D)** IRSs interact with RNA directly or through RNA-binding protein. This complex might control RNA metabolism.

There are a few papers reporting that IRSs are associated with various proteins. We have identified the proteins in IRSomes by yeast two-hybrid screening using IRSs as baits and LC-MS/MS analysis of proteins co-immunoprecipitated with IRSs. We succeeded in identifying at least 50 proteins that associate with IRSs in a tyrosine phosphorylation-independent manner. These complexes contain proteins modulating tyrosine phosphorylation of IRSs by receptor kinase, proteins controlling stability of IRSs, proteins determining intracellular localization of IRSs, and proteins mediating insulin-like activities. In addition, we found not only proteins that are involved in RNA metabolism but also RNAs themselves in these complexes (Figure 3).

#### Control of IRS Tyrosine Phosphorylation and Binding to PI3K

IRS-associated proteins can modulate IGF/insulin signal intensity through various mechanisms (Table 1; Figure 3A). One important role of IRS-associated proteins is to regulate the intensity of IGF/insulin signaling. IRSs are tyrosine-phosphorylated by the IGF-IR, IR, and other tyrosine kinases (117–123), resulting in activation of downstream signaling. Several tyrosine phosphatases are shown to dephosphorylate IRSs (124–128). The IRS dephosphorylation reaction can be modulated by other IRS-associated proteins. The mediator of ErbB2-driven cell motility 1 (MEMO1) binds to IRS-1 and competes with SH-PTP2 tyrosine phosphatase for IRS-1 interaction, thereby preventing IRS-1 dephosphorylation (129). SH2B1, a SH2 domain-containing adaptor protein, binds to IRS-1/2 and enhances insulin signaling by inhibiting tyrosine dephosphorylation of IRS-1/2 (130).

**Table 1:** **IRS-associated proteins that control IRS tyrosine phosphorylation and binding to PI3K**.

IRS-associated protein	IRS isoform	Function of IRS-associated protein	Reference
MEMO1	IRS-1	Inhibition of IRS-1 tyrosine dephosphorylation	(129)
SH2B1	IRS-1/IRS-2	Inhibition of IRS-1/2 tyrosine dephosphorylation	(130)
JIP1/2	IRS-1/IRS-2	Inhibition of IRS-1 tyrosine phosphorylation through mediating IRS serine/threonine phosphorylation by JNK	(131, 132)
Calmodulin	IRS-1/IRS-2	Inhibition of IRS-1 tyrosine phosphorylation	(133)
GKAP42	IRS-1	Enhancement of IRS-1 tyrosine phosphorylation by protecting the inhibitory effect of cGK-Iα	(134)
HSP90	IRS-2	Enhancement of IRS-2 tyrosine phosphorylation by mediating IRS-2 serine/threonine phosphorylation	(135)
HDAC2	IRS-1	Inhibition of IRS-1 tyrosine phosphorylation	(136)
Sirt1	IRS-1/IRS-2	Enhancement of IRS-2 tyrosine phosphorylation	(64, 137)
Pin1, Par14	IRS-1	Enhancement of IRS-1 tyrosine phosphorylation by IRS-1 proline isomerization	(138, 139)
Nedd4	IRS-2	Enhancement of IRS-2 tyrosine phosphorylation by IRS-2 mono-ubiquitination	(140)
Epsin1	IRS-2	Enhancement of IRS-2 tyrosine phosphorylation by the interaction of mono-ubiquitinated IRS-2	(140)
PHIP	IRS-1	Inhibition of IRS-1 tyrosine phosphorylation	(141)
ASPP2	IRS-1	Inhibition of IRS-1 tyrosine phosphorylation	(142)
14-3-3	IRS-1/IRS-2	Inhibition of IRS-1 tyrosine phosphorylation	(143, 144)
		Inhibition of the PI3K activity bound to IRS-1	(145)
		Other functions: Promoting of IRS-1 displacement from particular structures,	(146)
		and stabilization of IRS-2	(147)
Cytohesin-2/3	IRS-1	Enhancement of IRS-1 tyrosine phosphorylation possibly by facilitating the recruitment of IRS-1 to IR	(148)
Nexilin	IRS-1	Inhibition of IRS-1–PI3K binding	(149)

Many serine/threonine kinases are reported to phosphorylate IRSs at serine/threonine residues, which suppress IRS tyrosine phosphorylation (14). Some IRS-associated proteins modulate IRS tyrosine phosphorylation through the control of IRS serine/threonine phosphorylation. It is well known that JNK can induce IRS-1/2 serine phosphorylation, leading to the suppression of IRS tyrosine phosphorylation and insulin resistance (150). JIP1/2, JNK-interacting proteins, interact with IRS-1/2, and JIP deficiency causes increased IRS-1 tyrosine phosphorylation and abnormal glucose homeostasis in mice (131, 132), indicating that JIPs function as a scaffold linking IRSs and JNK. Calmodulin-dependent kinases (CAMKs) are known to induce IRS-1/2 serine phosphorylation (150), and the binding protein calmodulin interacts with IRS-1/2 (133). Calmodulin overexpression decreased insulin-induced IRS-1 tyrosine phosphorylation (133), suggesting that calmodulin may function as a scaffold linking IRSs and CAMKs. Recently, we identified GKAP42, a cGMP-dependent protein kinase (cGK) anchoring protein, as an IRS-1-associated protein (134). GKAP42 knockdown suppressed insulin-induced IRS-1 tyrosine phosphorylation, which was completely restored by the additional knockdown of cGK-Iα (134), suggesting that cGK-Iα inhibits IRS-1 tyrosine phosphorylation and GKAP42 protects the inhibitory effect of cGK-Iα. In some cases, IRS serine/threonine phosphorylation enhances IRS tyrosine phosphorylation. We reported that HSP90 interacts with IRS-2, and increases IRS-2 tyrosine phosphorylation through mediating IRS-2 serine/threonine phosphorylation by unknown kinase(s) in response to the activation of the cAMP pathway (135).

**Figure 4:**
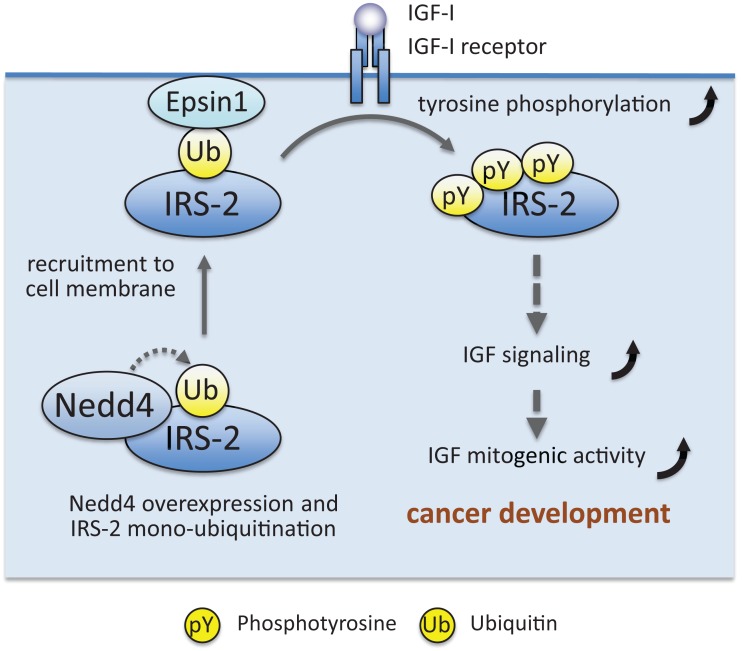
**Working model showing how Nedd4 enhances IGF signaling and mitogenic activity, leading to maintenance of cancer phenotype**. A detailed explanation is given in the text.

IRS-associated proteins can also affect IRS post-translational modifications other than serine/threonine phosphorylation. Histone deacetylase 2 (HDAC2) interacts with IRS-1, and overexpression of this protein suppresses insulin-induced IRS-1 tyrosine phosphorylation (136). Another deacetylase Sirt1 interacts with IRS-1 and IRS-2 (137), and inhibition of Sirt1 activity prevented deacetylation of IRS-2 and suppressed IRS-2 tyrosine phosphorylation (64, 137). Pin1 and Par14, peptidyl-prolyl *cis*/*trans* isomerases, interact with IRS-1 (138, 139). Pin1 enhances insulin-induced IRS-1 tyrosine phosphorylation through its isomerase activity, and Pin1 knockout mice exhibit impaired insulin signaling with glucose intolerance (138, 139). Similarly, Par14 enhances insulin-induced IRS-1 tyrosine phosphorylation, and Par14 knockdown exacerbated the glucose intolerance in Pin1 knockout mice (138, 139). Prolyl *cis*/*trans* isomerization in IRS-1 may be critical for efficient IRS-1 tyrosine phosphorylation. Recently, we found that ubiquitin ligase Nedd4 interacts with IRS-2 and induces IRS-2 ubiquitination (140). As described below, many ubiquitin ligases ubiquitinate IRS-1/2 and induce their proteasomal degradation, resulting in decreased IGF/insulin signaling. In contrast, Nedd4 increases IGF-I-dependent IRS-2 tyrosine phosphorylation and cell proliferation. Our subsequent study revealed that Nedd4 monoubiquitinates IRS-2, which promotes its association with Epsin1, a ubiquitin-binding protein localized at the plasma membrane. Nedd4 recruits IRS-2 to the plasma membrane, likely through promoting Epsin1 binding, and enhanced IRS-2 tyrosine phosphorylation by IGF-IR kinase (Figure 4). Nedd4 overexpression in zebrafish embryo accelerates the body growth and IRS-2 knockdown suppresses the effects of Nedd4 overexpression, indicating that Nedd4–IRS-2 complexes play important roles in body growth.

Some IRS-associated proteins can regulate the interaction between IGF-IR/IR and IRS, resulting in the modulation of IRS tyrosine phosphorylation. PHIP, a protein with largely unknown function, interacts with IRS-1 PH domain (141). ASPP2, a p53-binding protein, also interacts with the IRS-1 PTB domain (142). Overexpression of these proteins (or truncated proteins containing IRS binding sites) suppresses insulin-induced IRS tyrosine phosphorylation (141, 142). These observations support the hypothesis that these proteins impair the availability of IRSs to IR by binding to the PH/PTB domain. 14-3-3s interact with IRS-1/2 (143, 145, 151, 152) and suppress insulin-induced IRS-1 tyrosine phosphorylation possibly by inhibiting the interaction of the IRS-1 PTB domain with IR (143). Because 14-3-3–IRS association is dependent on PI3K pathway activation (145, 152), 14-3-3s may participate in the negative feedback regulation of IRS tyrosine phosphorylation. Other reports also show that 14-3-3 binding can modulate IRS-1 tyrosine phosphorylation through inhibiting the activity of PKCα (144), and can modulate the PI3K activity bound to IRS-1 (145), IRS-1 localization (146), as well as IRS-2 stability (147). Cytohesin-2/3, multi-domain proteins with guanine nucleotide exchange factor activity for ARF family GTPases, also interacts with IRS-1 and is proposed to facilitate the recruitment of IRS-1 to IR, thereby enhancing IRS-1 tyrosine phosphorylation (148).

An IRS-associated protein can modulate insulin signal intensity, not by changing IRS tyrosine phosphorylation but by affecting the interaction of IRS with the downstream effector. Nexilin, an actin binding protein, associates with IRS-1 under basal conditions, and this complex dissociates in response to insulin stimulation (149). Nexilin knockdown does not affect IRS-1 tyrosine phosphorylation but enhances IRS-1–PI3K interaction and Akt phosphorylation, whereas Nexilin overexpression inhibits Akt phosphorylation (149), suggesting that Nexilin suppresses IRS-1–PI3K interaction and the downstream insulin signaling.

#### Control of Stability of IRSs

Recent studies have shown that the stability of IRSs is controlled by IRS-associated E3 ligases and deubiquitinating enzymes (DUBs) via the ubiquitin-proteasome pathway (Table 2; Figure 3B). These E3 ligases and DUBs associate with IRSs under various conditions, resulting in dramatically changed IRS protein levels and induction of insulin-like activities according to surrounding environments.

**Table 2:** **IRS-associated proteins that control IRS protein stability**.

E3 ligase/DUB	IRS isoform	Stimulation	Tissues, cells	Mediated function	Reference
Cbl-b	IRS-1	Unloading stress (denervation and paceflight)	Skeletal muscles, C2C12 cells	Muscle atrophy	(153)
Cullin7-Fbxw8	IRS-1 (not IRS-2)	IGF-I	MEF, MCF-7 cells	Inhibition of oncogene- induced senescence	(59)
	IRS-1	Insulin	MEF, C2C12 cells	inhibition of insulin sensitivity in muscle	(154)
SCF-Fbxo40	IRS-1	IGF-I	Skeletal muscles, C2C12 myotube cells	Inhibition of body and muscle growth	(155)
Mdm2	IRS-1	Insulin	HIRc-B cells		(62)
MG53	IRS-1 (not IRS-2)	Skeletal myogenesis, obesity	Skeletal muscles, C2C12 myotube cells	Inhibition of skeletal myogenesis	(156, 157)
SOCS1/3	IRS-1/IRS-2	Insulin, inflammation	Liver, 3T3-L1 cells	Inflammation-induced insulin resistance	(156, 158–162)
USP7	IRS-1/IRS-2	IGF-I, insulin	FRTL-5, L6, H4IIE cells		(63)

It is well known that ubiquitination and degradation of IRSs are induced by prolonged IGF/insulin stimulation, which is one of the important mechanisms for the desensitization of IGF/insulin signaling (163–166). Several E3 ligases have been reported to induce the degradation such as cullin7 (Cul7)–Fbxw8 E3 ligase complex, SCF (Skp1, cullin1 and Rbx1)–Fbxo40 E3 ligase complex, SOCS1 and 3 complexes, and Mdm2 (59, 62, 155, 158). The degradation mechanisms of these E3 ligases were analyzed by using downstream pathway inhibitors such as PI3K inhibitor and ERK inhibitor; however, these results were often different. Some suggested that Cul7–Fbxw8 ubiquitinated and degraded IRS-1, but not IRS-2, via phosphorylation of IRS-1 by mTOR/S6K in MCF-7 cells (59), while ubiquitination by SCF–Fbxo40 required only direct signaling from IGF-IR in skeletal myotubes (155). And other reports suggested that activation of PI3K but not mTOR is required for IRS-1 degradation (167). In addition, it was reported that expression of Fbxo40 was muscle specific and was upregulated during differentiation, and the E3 ligases other than SCF–Fbxo40 did not regulate the IGF-I mediated degradation of IRS-1 in skeletal myotubes (155). These E3 ligases were also related to different bioactivities. Whereas embryonic fibroblasts of Cul7^−/−^ mice showed increased IRS-1 protein levels and activation of downstream Akt and MEK/ERK pathways, the null cells showed oncogene-induced senescence (59). And heterozygosity of either Cul7 or Fbxw8 increased PI3K/Akt activation in skeletal muscle tissue upon insulin stimulation, and these mice displayed enhanced insulin sensitivity and plasma glucose clearance (154). Knockdown of Fbxo40 also increased IRS-1 protein levels and activation of downstream pathways; however, knockdown of Fbxo40 induced hypertrophy of myofibers and Fbxo40^−/−^ mice showed enhanced body and muscle size during the growth phase unlike Cul7 (155). These observations suggested that each E3 ligase ubiquitinated IRSs via different signals and had different functions in specific tissues.

Other than IGF/insulin stimulation, various stimulation factors and physiological conditions affect IRS protein levels. So far, several E3 ligases had been identified as responsible factors for the degradation of IRS-1/2 under some conditions. Obesity and inflammatory cytokines secreted from adipose tissues induce IRS-1/2 degradation in insulin target tissues, which is thought to be one of the mechanisms for the onset of insulin resistance and type 2 diabetes. SOCS1/3 and E3 ligase MG53 expression, also known as TRIM72, were elevated by inflammation and obesity, which may induce insulin resistance in adipose tissue, liver, and skeletal muscle (156, 159, 160). Unloading stress such as denervation and spaceflight induces IRS-1 degradation and activates the FOXO3-dependent induction of atrogin-1/MAFbx in muscle, which causes unloading-mediated muscle atrophy. It was reported that unloading stress activated the E3 ligase Casitas B cell lymphoma-b (Cbl-b), and Cbl-b ubiquitinated and degraded IRS-1. And a pentapeptide mimetic of tyrosine 608-phosphorylated IRS-1 prevented the association with Cbl-b and IRS-1 and denervation-induced muscle atrophy in mice (153). Proper IRS-1 expression is important for IGF-induced myogenesis (168). MG53 is increased during myogenesis, and induces IRS-1 ubiquitination and degradation, thereby negatively regulating muscle differentiation (157).

Recently, we identified the deubiquitinating enzyme, ubiquitin-specific protease 7 (USP7) as an IRS-1/2 associated protein and showed that the deubiquitinase activity of USP7 plays important roles in IRS-1/2 stabilization through preventing their proteasomal degradation. In addition, IGF-I/insulin treatment dissociated USP7 from IRS-1/2 via the PI3K pathway activation (63). These findings suggested that the dissociation of USP7 from IRS-1/2 upon IGF/insulin stimulation may be involved in the feedback inhibition mechanism of IGF/insulin signaling by allowing the ubiquitination of IRS-1/2. Whereas protein degradation via the ubiquitin-proteasome pathway is regulated by the balance between ubiquitination and deubiquitination of target proteins, the understanding of DUBs remains less clear compared to E3 ligases. To elucidate the stability mechanism of IRSs, it is necessary to reveal not only how E3 ligases ubiquitinate IRSs but also how DUB(s) deubiquitinate IRSs. Taken together, these proteins controlling stability of IRS may present a novel approach for the treatment of diseases that are associated with insulin-like activities.

#### Control of Intracellular Localization of IRSs

IRS-associated proteins can control IRS intracellular localization (Table 3; Figure 3C). It has been suggested that tyrosine phosphorylation of IRSs occurs at the cell surface because IGF-IR/IR is activated at the cell surface. Despite this claim, early investigations using subcellular fractionation revealed that phosphorylated IRS and its associated PI3K activity are abundant in the intracellular membrane compartments but less so in the plasma membrane (169–171). Another observation was that other growth factors such as platelet-derived growth factor (PDGF) that equally activate PI3K do not significantly induce glucose uptake. Since PDGF stimulates PI3K mainly in the plasma membrane (172), the IRS localization to intracellular membrane compartments is thought to be an important component of IGF/insulin-specific actions. However, IRSs are cytosolic proteins without post-translational modification that target it to the membrane structures. Thus, they rely instead on interaction with membrane-associated factors (Table 3; Figure 3C). Two clathrin adaptor complexes, AP-1 and AP-3, have been reported to regulate the subcellular localization of IRS-1 (173, 174). It is well established that clathrin mediates the intracellular trafficking of cargos between different membrane compartments through the formation of the coated-vesicles (175). AP complexes are heterotetrameric and function in cargo selectivity and the initiation of clathrin vesicle formation (176). In particular, AP-1 sorts cargo proteins between endosomes and the *trans*-Golgi network, whereas AP-3 is involved in the transport to lysosomes or related organelles. AP-1 interacts with IRS-1 through its μ1A subunit, which recognizes the YxxΦ-type motifs in the cargo proteins. While IRS-1 is predominantly localized to vesicular structures, an IRS-1 mutant lacking the binding sites to μ1A is mislocalized to the late endosomes that are positive for cation-independent mannose-6-phosphate receptor, suggesting that IRS-1 is transported from late endosomes to peripheral vesicles through the interaction with the AP-1 complex. Furthermore, depletion of AP-1 binding sites in IRS-1 impaired IGF-I-induced cell proliferation, accompanied by reduced IRS-1–PI3K signaling. Interestingly, AP-1 binding sites in IRS-1 overlap PI3K binding sites when phosphorylated by IGF-IR/IR (55, 177). By contrast, it has been structurally demonstrated that AP-1 cannot bind to the phosphorylated YxxΦ motif (178). These observations offer a model in which IRS-1 is sorted by AP-1 and subsequently dissociates from AP-1, and then is targeted for IGF/insulin-mediated tyrosine phosphorylation and downstream signaling activation in a spatially compartmentalized manner. IRS-1 also interacts with σ3 subunit of AP-3 (174). σ3 has been shown to possess an activity to translocate IRS-1 from the cytosol to membrane fractions *in vitro*. From the investigations of other AP complexes, AP-1 and AP-2, σ3 together with the δ subunit is expected to recognize the di-leucine motif (179), implying that the interaction mode with AP-2 is different from that with AP-1.

**Table 3:** **Proteins involved in the subcellular localization of IRS**.

IRS-associated protein	IRS isoform	Function of IRS-associated protein	Reference
μ1A (AP-1 complex)	IRS-1	Sorting IRS-1 to the intracellular vesicles through AP-1 complex	(173)
σ3 (AP-3 complex)	IRS-1	Translocating IRS-1 to the membrane fraction *in vitro*	(174)
APPL1	IRS-1/IRS-2	Recruitment of IRS-1/IRS-2 to insulin receptor	(180)
SV40/JCV T-antigen	IRS-1	Targeting IRS-1 to nucleus	(181, 182)
Importin β	IRS-3	Targeting IRS-3 to nucleus	(183)
Caveolin-1	IRS-1	Recruitment of IRS-1 to caveolae (proposed)	(184)

Components other than AP-1 and AP-3 complexes have been reported to regulate the targeting of IRS-1 to the intracellular membrane compartments (Table 3). Early investigation of the IRS-1-enriched membrane fraction using electron microscopy implied that IRS-1 is localized to the intracellular membrane compartments through the cytoskeletal proteins such as actin (169, 185). Recently, IRS-1 and IRS-2 have been reported to interact with APPL1, which regulates the endocytosis from plasma membrane to early endosomes (180). APPL1 also interacts with IR and deletion of APPL1 reduced the IR-IRS signaling, suggesting that APPL1 serves as the platform for the interaction between IRS and the receptor. Given that IRS localized in endosomes is phosphorylated, IGF-IR/IR should be internalized after ligand stimulation. In fact, several reports suggest that internalization of IGF-IR/IR is required for proper signaling (186–189). How the trafficking of IGF-IR/IR and IRS localization are coordinated to activate the downstream signaling remains an open question that needs to be addressed in further studies.

In addition to the targeting to the intracellular membrane compartment, IRSs have been reported to localize to other sites in the cells. For example, IRS-1 has been found in nucleus (181, 182, 190). Nuclear targeting of IRS-1 is mediated by the interaction with viral T-antigens such as SV40 and its human equivalent (181, 182). Furthermore, nuclear IRS-1 is suggested to function in ribosomal RNA (rRNA) biosynthesis and the transcription of cell cycle genes such as c-myc and cyclin D1 (191, 192). Nuclear IRS-1 is also reported to interact with Yes-associated protein (YAP1), an oncogenic transcription factor negatively regulated by the Hippo pathway (193). IRS-3, another IRS family protein, is also localized to the nucleus (194). IRS-3 interacts with importin β, which together with importin α mediates the protein import through the nuclear pore complex (183). *In vitro* binding analyses revealed that the PTB domain of IRS-3 interacts with importin β, contains the sequence required for its nuclear targeting (183, 194), and is not conserved in those of IRS-1 and IRS-2, both of which are predominantly localized to the cytosolic portion. In addition, it has been shown using Gal4 chimeric transcription assay that among IRS proteins, only IRS-3 possesses the transcriptional activity (194). This activity partially accounted for the evidence that IRS-3 interacts with Bcl-3, which forms a complex with p50 NF-κB, and enhances NF-κB-dependent anti-apoptotic gene expression (84).

As mentioned above, there is the possibility that the tyrosine phosphorylation of IRSs in the plasma membrane does not contribute to the downstream signaling. However, several reports suggested that IRS localizing at specific regions of the plasma membrane called caveolae functions in insulin signaling. Caveolae are the invaginated microdomains of the plasma membrane enriched in cholesterol and sphingolipids. Both IR and IRS-1 were found in caveolae in 3T3-L1 adipocytes (16, 195, 196). Among the various components of caveolae, IRS-1 has been shown to interact with caveolin-1 (184). Investigations using electron microscopy revealed that IR is concentrated in the neck of caveolae in response to insulin (195, 197), suggesting that the caveolae function as microdomains for insulin signal transduction. The primary cilium has been recently shown as another microdomain in the plasma membrane where a portion of IRS-1 is targeted (198). Phosphorylation of a portion of IRS-1 and Akt was found in the cilia, indicating that the IRS-mediated signaling is activated at the cilia. Interestingly, HSP90, which is known to interact with IRS-2 (135), was identified as a chaperone that forms the neck of the cilia, implying that HSP90 represents IGF/insulin signaling platform in the cilia.

#### Control of RNA Metabolism and Translation

In seeking to identify IRS-interacting proteins, we found that proteins related to RNA translation, such as poly(A) binding protein (PABP), and eukaryotic initiation factors (eIF) 4E and 4G, associate with IRS-1 in MCF-7 human breast cancer cells (88) (Table 4; Figure 3D). Moreover, the formation of the high-molecular-mass complex containing IRS-1 (IRS-1 complex) is abolished by treatment with RNase but not the IRS-2 complex, indicating that only the IRS-1 complex contains RNAs. Indeed, we found that poly(A)+ RNAs are contained in the IRS-1 complex, and the interactions between IRS-1 and PABP/eIF4E/4G are mediated by RNA (88). Although IGF and insulin have been shown to activate protein synthesis through the IRS-1–mTORC1 pathway (199), it has not yet been reported that the messenger ribonucleoprotein (mRNP) complex included IRS-1. Notably, in the absence of growth factors, the IRS-1 complex seems to associate with monosomes, but in the highly proliferating cells that are stimulated with serum, IRS-1 complexes increase their molecular mass associating with polysomes (88). Together, these findings suggest that RNA-containing IRS-1 complexes have some function in the cells, which raises a new possibility that IRS-1 plays an unrevealed role as a platform for the translation of mRNA species.

**Table 4:** **IRS-associated proteins that are involved in RNA metabolism**.

IRS-associated protein	IRS isoforms	Functions in the RNA metabolism	Reference
eIF4E	IRS-1	Involved in directing ribosomes to the cap structure of mRNAs at the step of protein synthesis	(88)
eIF4G	IRS-1	Scaffold protein involved in the formation of the eIF4F complex on the cap structure of mRNA	(88)
PABPC1	IRS-1	Required for the 3′-poly(A) tail shortening and translation initiation of mRNA	(88)
SAM68	IRS-1	Alternative splicing regulator whose function depends on its protein modification in response to extracellular cues	(200)

In addition, IRS-1 could regulate the translation of mRNAs surrounding itself; however, the mechanism of the activation requires much additional investigation. Further, the specific mRNA regulated by this complex needs to be established for full appreciation of this new model of IRS-1 action. It will be also important to reveal how mRNAs are recruited to the IRS-1 complex. Since IRSs do not have a canonical RNA-binding domain, it is possible that some undefined RNA-binding proteins are contained in IRS-1 complex and these proteins tether the selected mRNAs to IRS-1.

In relation to the novel possible function of IRS-1 in mRNA translation, as estimated by the interaction of IRS-1 with mRNP components like PABP, eIF4E, and eIF4G (88), we found that IRS-1 also forms a complex with U96A small nucleolar RNA (snoRNA) (89), which functions in the post-transcriptional rRNA modification in the nucleus (201, 202). Cross-linking immunoprecipitation (CLIP), which is a method to analyze protein–RNA interaction, revealed that IRS-1 somehow interacts with U96A snoRNA in close proximity (89). Moreover, the amount of U96A snoRNA is lower in the IRS-1-deficient cells compared to wild-type cells, which suggests that IRS-1 plays a role in the snoRNA biogenesis. Since modification of rRNA has been shown to be required for a specific class of mRNA translation, such as internal ribosome entry site (IRES) containing mRNA (203, 204) in addition to the translation of the general mRNA translation (205), it is possible that IRS-1 takes part in upregulating a specific type of snoRNA abundance in cells, which results in the sequential rRNA maturation and ribosome function in the selected mRNA translation as well. Previous studies have been shown that IRSs are localized in the nucleus under various conditions (181, 190, 206, 207), and function in the expression of genes encoding the factors that drive cell proliferation including rRNAs (191, 208). Thus, IRS-1 seems to function in both transcription and modification of rRNA, thereby regulating cell proliferation in a multilayer fashion. Determining precisely how IRS-1 localizes in the nucleus and binds to snoRNA is an essential step to further the understanding of these complex interactions.

As described in the sections above, the proteins that interact with IRSs determine their phosphorylation status, stability, and intracellular localizations. It is not clear, however, if all their functions are induced simultaneously in the same IRS–RNA complex or if individual complexes specifically modulate the IRS-mediated RNA translation. Further analysis is needed to resolve these issues; however, the ability of IRSs to control RNA metabolism and translation as well as the signaling outcomes via differences in the association with their partners is a fascinating new model of IGF/insulin-mediated protein synthesis.

### Possible functions of IRSome in physiology and diseases including cancer

A lot of IRS-associated proteins are related to the physiological regulation of insulin-like activity. As described above, IGF bioactivity is often potentiated by other hormones, by which appropriate IGF activities will be expressed *in vivo* (209). In thyroid epithelial cells, TSH stimulation and the following activation of cAMP pathway enhance IGF-I-induced IRS-2 tyrosine phosphorylation and cell proliferation (209), which is thought to be important for thyroid morphogenesis and thyroid hormone homeostasis (210). We reported that HSP90 and Nedd4 mediate cAMP-dependent enhancement of IRS-2 tyrosine phosphorylation and cell proliferation (135, 140). Notably, Nedd4–IRS-2 interaction is one of the important tuning points in this signal crosstalk, because Nedd4–IRS-2 interaction is upregulated in response to cAMP and it triggers the mechanisms by which cAMP signaling enhances IGF signaling (140). Conversely, IGF/insulin signaling can modulate other signaling pathways through IRS-associated proteins. IRS-1 interacts with the inhibitory G protein Gi in response to insulin in platelets, which may enhance cAMP signaling and lead to the attenuation of platelet activation (211). IRS-1/2 interacts with and stabilizes Disheveled, thereby promoting Wnt/beta-catenin signaling (212). IRS-1/2 interacts with sarco-endoplasmic reticulum calcium ATPase (SERCA), and may regulate Ca^2+^ signaling (213, 214). IRS-1/2 can also interact with Bcl-2, and may modulate apoptosis signaling (215).

IRS-associated proteins are relevant to insulin resistance and abnormal glucose metabolism. Obesity increases inflammatory cytokines including TNF-α. TNF-α decreases GKAP42, an IRS-associated protein that enhances IRS-1-mediated signaling (134), and also induces the association of IRS-1 with calmodulin that suppresses IRS-1-mediated signaling (133, 216). Cytokine-induced JNK activation causes IRS-1 serine phosphorylation and impairs insulin signaling, which is mediated by IRS-associated protein JIP (131, 132). Obesity and inflammatory cytokines increase ubiquitin ligase SOCS1/3 and MG53, thereby decreasing IRS-1/2 levels (156, 159, 160). Obesity seems to impair APPL1 function as a linker of IRSs to IR (180). These events could contribute to the induction of insulin resistance and diabetes. On the other hand, food intake increases Pin1, an IRS-associated protein that enhances IRS-1-mediated signaling, thereby leading to increased insulin sensitivity and adiposity (138). Several Sirt1 activators have beneficial effects on glucose homeostasis and insulin sensitivity in obese mice (217), which is partly explained by the function of Sirt1 as an IRS-associated protein that enhances IRS-2-mediated signaling (64, 137).

IRS-associated proteins may also contribute to the maintenance of the cancer phenotype. Nedd4 and Epsin1, which coordinately enhance IGF-I-induced IRS-2 tyrosine phosphorylation, are often overexpressed in cancer cells (218, 219). Nedd4 is known to function as an oncogenic protein (218), and we showed that Nedd4 is required for IGF-I-induced proliferation of prostate cancer cells (140) (Figure 4). Another IRS-associated protein MEMO1 is often increased in aggressive cancers, and is reported to induce epithelial–mesenchymal transition in breast cancer cells and enhance their metastasis. The underlying mechanism partly involves preventing IRS-1 dephosphorylation and increasing IGF signaling (129). Protein levels of IRSs are often upregulated in various cancer cells. IRS-associated ubiquitin ligases and DUBs may contribute to this upregulation. Nuclear IRSs are also often observed in cancer, and many IRS-associated proteins are suggested to mediate cancer-related nuclear IRS functions (114). T-antigens, v-src, and estrogen receptors can interact with IRSs and mediate IRS nuclear translocation. In nuclei, Rad51 recombinase interacts with IRSs, and the interaction is proposed to impair DNA repair. UBF-1, a key regulator of RNA polymerase-I, also interacts with nuclear IRSs, and the interaction is proposed to enhance rRNA synthesis, thereby promoting cell growth. A transcriptional factor β-catenin also interacts with nuclear IRSs, which enhance the expression of the β-catenin target genes that promote cell proliferation. Through these interactions, nuclear IRSs may participate in cancer development (114). In addition, as described above, IRSs possess cell transformation promoting ability. Although the molecular mechanism how IRSs promote cell transformation remains largely unclear, the observation that IRS can interact with oncoproteins including SV40 T-antigen, JCV T-antigen, Ret, NPM–ALK, and ETV6–NTRK3 (122, 182, 220–223) provide clues to the mechanism.

## Conclusion

We and others discovered that IRSs are associated with a variety of proteins. IRSs form high-molecular-mass complexes even without their tyrosine phosphorylation (Figure 1C). These complexes contain proteins modulating insulin-like signals of IRSs triggered by receptor tyrosine kinase, proteins controlling stability of IRSs, proteins determining intracellular localization of IRSs, proteins involved in RNA metabolism, and RNAs themselves (Figure 3). Based on these results, we propose that IRSs function not only as signaling mediators through tyrosine phosphorylation but also as scaffold proteins independent of tyrosine phosphorylation in mediation of novel biological activities or modulation of insulin-like activities (Figures 1 and 3).

However, these proteins seem not to function independently. In other words, they are likely to interlock. For example, after IRSs are synthesized, IRSs are recognized by sorting proteins and recruited to specific intracellular compartments. IRSs are associated with the specific proteins there. Another example is intramolecular modification of IRSs. IRSs are post-translationally modified by IRS-associated proteins, such as serine/threonine kinases or phosphatases, E3 ubiquitin ligases or DUBs, acetylases or deacetylases, which are activated by various extracellular factors, such as hormones and cytokines and various physiological states. And then, differentially modified IRSs are recognized by different IRSAPs, which mediate novel biological activities or modulate insulin-like activities. For these reasons, the phenotypes of mice or cells in which IRSs were knocked out or knocked down should be reconsidered from the point of view of IRS-associated proteins.

As mentioned in the Introduction, when dysregulation of insulin-like activities is induced, many types of age-related diseases including diabetes, cancer, neurodegenerative disease, arteriosclerosis, and osteoporosis will develop (12–14). Based on our and others’ recent data about IRSAPs, IRSAPs would be expected to contribute to the modulation of insulin-like activities. Thus, the disorder of modulation of insulin-like activities that underlies several age-related diseases including cancer is possibly explained by IRSome function. The IRSome is an important new target for the treatment of these diseases by controlling insulin-like activities.

## Author Contributions

Wrote, read, and approved the final manuscript: FH, TF, YY, HK, AO, HY, DY, TS, MS-Y, BY, KC, S-IT.

## Conflict of Interest Statement

The authors declare that the research was conducted in the absence of any commercial or financial relationships that could be construed as a potential conflict of interest.

## Abbreviations

BN-PAGE, blue native-polyacrylamide gel electrophoresis; CLIP, UV cross-linking and immunoprecipitation; IGF-IR, IGF-I receptor; IGFs, insulin-like growth factors; IR, insulin receptor; IRES, internal ribosome entry site; IRSAPs, IRS-associated proteins; IRSome, high-molecular-mass complexes containing IRSs; IRSs, insulin receptor substrates; mRNP, messenger ribonucleoprotein; PH, pleckstrin homology; PI3K, phosphoinositide 3-kinase; PTB, phosphotyrosine binding; rRNA, ribosomal RNA; SH, Src-homology; snoRNA, small nucleolar RNA; TNF, tumor necrosis factor; TSH, thyrotropin.
